# (5R)-5-Hydroxytriptolide (LLDT-8) induces substantial epigenetic mediated immune response network changes in fibroblast-like synoviocytes from rheumatoid arthritis patients

**DOI:** 10.1038/s41598-019-47411-1

**Published:** 2019-08-01

**Authors:** Shicheng Guo, Jia Liu, Ting Jiang, Dungyang Lee, Rongsheng Wang, Xinpeng Zhou, Yehua Jin, Yi Shen, Yan Wang, Fengmin Bai, Qin Ding, Grace Wang, Jianyong Zhang, Xiaodong Zhou, Steven J. Schrodi, Dongyi He

**Affiliations:** 1 Center for Precision Medicine Research, Marshfield Clinic Research Institute, Marshfield, WI United States 54449; 2 Department of Rheumatology, Shanghai Guanghua Hospital of Integrated Traditional and Western Medicine, Shanghai, 200052 China; 3 Arthritis Institute of integrated Traditional and Western medicine, Shanghai Chinese Medicine Research Institute, Shanghai, 200052 China; 4 Division of Biostatistics, University of Texas School of Public Health, Houston, TX USA; 5 Washington University, St. Louis, Missouri 63130 USA; 6 Shenzhen Traditional Chinese Medicine Hospital and The fourth Clinical Medical College of Guangzhou University of Chinese Medicine. Fuhua Road, Shenzhen, Guangzhou, 518033 China; 7 University of Texas Medical School at Houston, 6431 Fannin, MSB5.270, Houston, TX 77030 USA; 8 Computation and Informatics in Biology and Medicine, University of Wisconsin-Madison, Madison, WI 53706 USA

**Keywords:** Immunogenetics, Rheumatoid arthritis

## Abstract

*Tripterygium* is a traditional Chinese medicine that has widely been used in the treatment of rheumatic disease. (5R)-5-hydroxytriptolide (LLDT-8) is an extracted compound from *Tripterygium*, which has been shown to have lower cytotoxicity and relatively higher immunosuppressive activity when compared to *Tripterygium*. However, our understanding of LLDT-8-induced epigenomic impact and overall regulatory changes in key cell types remains limited. Doing so will provide critically important mechanistic information about how LLDT-8 wields its immunosuppressive activity. The purpose of this study was to assess the effects of LLDT-8 on transcriptome including mRNAs and long non-coding RNA (lncRNAs) in rheumatoid arthritis (RA) fibroblast-like synoviocytes (FLS) by a custom genome-wide microarray assay. Significant differential expressed genes were validated by QPCR. Our work shows that 394 genes (281 down- and 113 up-regulated) were significantly differentially expressed in FLS responding to the treatment of LLDT-8. KEGG pathway analysis showed 20 pathways were significantly enriched and the most significantly enriched pathways were relevant to Immune reaction, including cytokine-cytokine receptor interaction (*P* = 4.61 × 10^−13^), chemokine signaling pathway (*P* = 1.01 × 10^−5^) and TNF signaling pathway (*P* = 2.79 × 10^−4^). Furthermore, we identified 618 highly negatively correlated lncRNA-mRNA pairs from the selected significantly differential lncRNA and mRNA including 27 cis-regulated and 591 trans-regulated lncRNA-mRNAs modules. KEGG and GO based function analysis to differential lncRNA also shown the enrichment of immune response. Finally, lncRNA-transcription factor (TF) and lncRNA-TF-mRNA co-expression network were constructed with high specific network characteristics, indicating LLDT-8 would influence the expression network within the whole FLS cells. The results indicated that the LLDT-8 would mainly influence the FLS cells systemically and specially in the process of immune related pathways.

## Introduction

Rheumatoid arthritis (RA) is the most common chronic inflammatory disease with complex etiology^1^. The inflammation makes the synovium thicken and causes swelling around the joints. Thus, it induces the damage of cartilage. Recent findings indicate that remission of symptoms is more likely when treatment begins early with medications^2^. Although several widely-used anti-rheumatic drugs, such as methotrexate, steroids, anti-IL-6R and anti-TNFa monoclonal antibodies, have demonstrated some degree of efficacy in the treatment of RA, a substantial proportion of patients exhibit poor response and the incidence of adverse reactions is substantial. The use of plant- and microbial-based compounds to treat a variety of pathologies has an extensive history and includes digitalis, L-Dopa, taxol, quinine, ephedrine, codeine, and penicillin^3,4^. In our clinical practice, we found a traditional Chinese medicine *Tripterygium* could be applied to decrease the severity of RA. *Tripterygium* is a genus of plants from the Euonymus Corey Gong. This Traditional Chinese medicine has been used in the treatment of autoimmune disease^5,6^, rheumatic disease^7,8^, and systemic lupus erythematosus (SLE)^9,10^. Triptolide is an extracted compound from *Tripterygium*, which has strong immunosuppressive activity. Triptolide can inhibit interleukin-6 (IL-6) and reduce osteoclastogenesis by inhibiting NF-κB signaling. An optimized structured analog of Triptolide, (5R)-5-hydroxytriptolide (LLDT-8), has been shown to have both low toxicity and high immunosuppressive ability^11^. Previous studies also demonstrated that LLDT-8 can suppress the immune responses on peripheral blood mononuclear cells (PBMC) and T cells^11^. Importantly, LLDT-8 also inhibited the differentiation of Th1 and Th17 cells and has effect on the immune responses of RA patients^12^. Increasing evidence indicates that activated synovial fibroblasts, together with macrophage and lymphocyte secreted factors, as part of a complex cellular network, play an important role in the pathogenesis of rheumatoid arthritis^13^. Meanwhile the effect of LLDT-8 to cells have been also validated in our previous report^11^ and other studies *in vivo*^14^ and *in vitro* experiments^15–19^. However, the pharmacological effect of LLDT-8 on synovial fibroblasts has not been investigated. Further, elucidating the underlying molecular mechanisms of how LLDT-8 generates immunosuppression without a high degree of toxicity may provide a platform for the development of novel anti-rheumatologic therapeutics.

Long non-coding RNAs (lncRNAs) are a recently discovered class of non-coding functional RNA and some important roles of lncRNAs as regulators in a wide spectrum of biological processes were identified in the past several years^20–22^. There are over 16,000 lncRNAs in humans and the functions of majority of them are unknown. Recent evidence also suggests the role of lncRNAs in the pathophysiology of disease processes, especially in cancer and immune disease^23–27^. In the previous studies, lncRNA have been reported to be related to the pathogenesis of RA due to its association of major pathways linked to Inflammation, such as NFkB and TLR signaling^26^. However, whether lncRNA is involved in the mechanism of therapy of LLDT-8 to rheumatoid arthritis is still unknown.

The aim of this study was designed to identify the mechanism of immuno-impressive function of LLDT-8 and identify potential ncRNA targets of LLDT-8. We applied TNF-α and IL-17 to induce an inflammatory status in the cells so that we can observe the LLDT-8 effect easily compared with treatment to normal FLS cell directly. We then assessed the effects of LLDT-8 on the regulation of gene expression on mRNAs and long non-coding RNAs in FLS isolated from RA patients. Agilent Human lncRNA (4 × 180 K, Design ID: 062918) was applied in present study to provide genome-wide ncRNA and mRNA expression profile. With this study, we aimed to demonstrate how LLDT-8 treatment significantly impacts the process of immune regulation.

## Material and Methods

### Patients, cell culture and LLDT-8 treatment

FLS cells were derived from the synovial tissues of patients with RA in Guanghua hospital who had undergone total joint replacement surgery during June to August 2015. Written informed consent to collect FLS cells for LLDT-8 studies was obtained from all participating RA patients. This study was approved by the academic advisory board of Guanghua Hospital (No. 2015-SRA-01) and all methods were performed in accordance with the relevant guidelines and regulations. All patients were random enrolled and all of them fulfilled the American College of Rheumatology classification criteria for RA^28^. Clinical data were collected at the time of sample collection. The detail clinical information is shown in Table 1.

**Table 1:** Clinical and demographical Characteristics of 5 RA patients.

ID	COD(Year)	SJC	TJC	ESR	CRP	PGA	DAS28-CRP
RA-001	5	4	4	19	29.8	40	4.3
RA-002	20	10	10	27	25.97	70	5.66
RA-003	6	1	1	25	6.1	60	3.58
RA-004	8	1	2	11	1.3	60	3.05
RA-005	20	18	16	21	18	65	6.19

COD: course of a disease; SJC: swollen joint count; TJC: tender joint count; ESR: erythrocyte sedimentation rate; PGA: patient global assessment; DAS28-CRP: disease activity score in 28 Joints.

**Figure 1:**
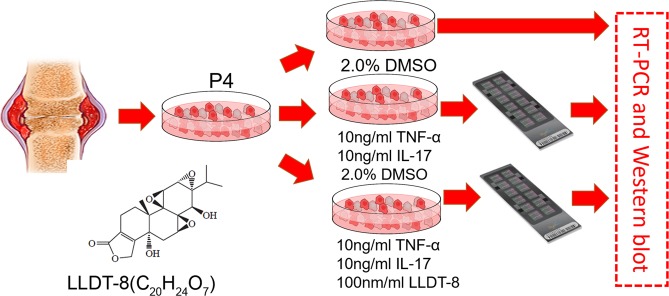
Flowchart of the study. In the study, we collected 5 RA joint tissues from joint replacement surgery. FLS cells were purified with cell culture and validated by flow cytometry assay. When cells approached confluence, they were passed after diluting 1:3 with fresh medium until used. The purity of the cells was verified by flow cytometric analysis. The FLS, from passages 4, were seeded in 6-well plates. As the control group, the FLS cells were cultured in DMEM including 10 ng/ml TNF-α and 10 ng/ml IL-17 (PeproTech, USA) for 12 h. In the treatment group, LLDT-8 (Shanghai Pharma, Shanghai) was dissolved with 2% DMSO and diluted with DMEM (with TNF-α and IL-17) to 100 nM for 24 hours. RT-PCR, Western blot and microarray were then conducted to different groups.

An overview of the cellular processing is shown in Fig. 1. Briefly, joint tissues were minced into 1 × 1 × 1 mm pieces and treated for 2 hours (h) with 2–4 mg/ml of collagenase (Serva, GERMANY) in DMEM at 37 °C in 5% CO2. Dissociated cells were then centrifuged at 500 × g, re-suspended in DMEM supplemented with 10% FCS (Gibco, USA), and plated in 75 cm^2^ flasks. The cultures were kept at 37 °C in 5% CO2 and the culture medium was replaced every 2–3 days. Mycoplasma assay was performed on synovial fibroblasts to avoid mycoplasma contamination. When cells approached confluence, they were passed after diluting 1:3 with fresh medium until used. The purity of the cells was verified by flow cytometric analysis. The FLS cells of passages 4th were seeded in 6-well plates. As the control group, the FLS cells were cultured in DMEM including 10 ng/ml TNF-α and 10 ng/ml IL-17 (PeproTech, USA) for 12 h. In the treatment group, LLDT-8 (Shanghai Pharma, Shanghai) was dissolved with 2% DMSO and diluted with DMEM (with TNF-α and IL-17) to 100 nm/ml for 24 hours. 100 nM LLDT-8 does not have obvious FLS cytotoxic effect while producing a slight apoptosis effect for FLS (Supplementary Fig. 1) which is consistent with a previous report^11,29^. In the process to validate the microarray data, TNF-α treatment was applied to simulate a pro-inflammatory reaction and then LLDT8 treatment was applied to evaluate the effect of LLDT-8 on the inflammatory reaction as measured by real-time PCR (RT-PCR).

### RNA extraction and microarray hybridization

The total RNA was extracted by TRIzol reagent and quantified by the NanoDrop ND-2000 (Thermo Scientific, Waltham, MA, USA). The integrity of RNA was assessed using the Agilent Bioanalyzer 2100 (Agilent Technologies Inc, Santa Clara, CA, USA).

Customized Agilent Human lncRNA microarray (4 × 180 K, Design ID: 062918) was used in this study. 30,656 probes located in mRNA transcripts from Entrez Gene and 78,243 probes in lncRNA. the catalog of the lncRNA were collected from the integration of Broad Institute, Human Body Map lncRNA, TUCP catalog, UCSC lncRNA Transcripts, GENCODE 18, NONCODE V4.0, Ensembl, RefSeq, Ultra-conserved region encoding LncRNA (UCR), lncRNAdb and ncRNA database. The total RNA was transcribed to double-stranded cDNA, synthesized into cRNA (Low Input Quick-Amp Labeling Kit, one-color, Agilent), and labeled with Cyanine-3-CTP. The labeled cRNAs were then hybridized onto the microarray. After washing, the arrays were scanned by the Agilent Scanner G2505C (Agilent Technologies Inc., Santa Clara, CA, USA). Other parameters in ncRNA labeling, microarray hybridization, and washing procedure were performed according to the manufacturer’s standard protocols. RT-PCR was applied to validate parts of significant differential genes within same samples with > 3 times technical repeats.

### Image scanning and data analysis

The array images were analyzed using Feature Extraction software (version 10.7.1.1, Agilent Technologies Inc., Santa Clara, CA, USA), and the raw data were obtained and basically analyzed with Genespring. The raw data were initially normalized with the quantile algorithm. The probes that at least in one out of two conditions had flags in “P” were chosen for further data analysis. Differentially expressed genes or lncRNAs were identified through a combination of fold change and p-values calculated from a t-test (details in the Statistical analysis section). The threshold set for upregulated and downregulated genes was a fold change ≥ 2.0 and a P ≤ 0.05.

### Identification of cis-regulated mRNAs of the differential lncRNAs

Based on these 5 biological replications with or without LLDT-8 treatment, differential expressed lncRNA, cis-regulatory genes/mRNAs were identified. The mRNAs were identified as “cis-regulated mRNAs” when (1) the mRNA loci are within 100 k windows up- and down-stream of the given lncRNA, and (2) the Pearson correlation of lncRNA–mRNA expression is significant (P ≤ 0.01). GO analysis and KEGG analysis were applied to determine the roles of these differentially expressed mRNAs. Finally, Hierarchical Clustering was performed to display the distinguishable genes’ expression pattern among samples.

### Functional prediction of selected differential lncRNAs

The overall gene function distribution (co-expression, ontology and pathway) of the differential lncRNAs obtained in the experiment was identified as follows. For each differential lncRNA, the Pearson correlation of its expression value with the expression value of each mRNA was calculated, and a P < 0.05 was selected. The enrichment of functional terms in annotation of differential expressing gene or co-expressed mRNAs was statistically evaluated using the hypergeometric cumulative distribution function^30,31^.

### Identification of transcription factors (TF) associated to differential lncRNAs

The transcription factor/chromatin regulation complexes that may possibly play a co-regulatory role with lncRNAs were identified^32,33^. In brief, the set between the lncRNA co-expression coding genes and the target genes of transcription factors/chromatin regulation complex was collected respectively. The enrichment level of the set was determined using hypergeometric distribution, thus the transcription factors significantly associated with differential lncRNAs were finally screened. Finally, the co-expression networks among lncRNA, TF, and target genes were built with Cytoscape 3.4.0^34^.

### Western Blot and Immunofluorescence

Western Blot and Immunofluorescence were applied to detect the protein expression change and nuclear localization to show the effect of LLDT-8 treatment to RF cells in our validation stage and the procedures are same as the previous study^2^. Briefly, FLS cells were seeded at a density of 1 × 10^7^ cells per well (6-well plates) in DMEM supplemented with 10% FBS and 1% penicillin. After cell attachment, the culture medium was replaced by DMEM supplemented with A panel: DMSO (2%), B panel: TNF-α (10 ng/ml) and IL-17 (10 ng/ml) or C panel: TNF-α (10 ng/ml), IL-17 (10 ng/ml) and LLDT-8 (0, 50 nM, 100 nm, respectively) for 12 hours. The cells were collected and lysed by 1 nM PMSF SDS on ice for 30 min. The cellular lysates were loaded, and proteins were separated on SDS-PAGE and transferred to nitrocellulose filter. The blots were blocked with 5% BSA TBST for 1 h at room temperature, then probed with rabbit anti-mouse antibodies against p-P65(1:1000) and GAPDH (1:6000) overnight at 4 °C. After five washes, the blots were subsequently incubated with a HRP-linked secondary antibody (1:2500) for 90 min at room temperature. The blots were visualized by ECLTM Prime Western Blotting Detection Reagent. FLS with different treatments were fixed using 4% (v/v) formaldehyde in PBS for 15 min and mounted onto slides using VECTASHIELD Antifade Mounting Medium containing DAPI (Vector Laboratories, H-1200) and visualized using an inverted SP5 confocal microscope (Leica).

### Statistical analysis

One sample t-test was applied to compare the mean difference of the expressed level before and after the treatment of LLDT-8. All the statistical analysis was made using R version 3.2.2. We selected the top signals using the threshold on both the fold change > 2 and the P-value < 0.05 from mRNA and lncRNA. In addition, we calculated the Pearson correlation for the expression values on every different combination of lncRNA and mRNA. The pair of IncRNA-mRNA will be selected if their correlation test reaches the threshold (P-value < 0.05). Also, in order to ensure the selected pairs are meaningful, we only selected the correlated pairs in which lncRNA plays as the inhibition of mRNA expression (correlation coefficient, *r* < −0.9). False discover rate (FDR) correction was performed by Benjamini-Hochberg procedure with default R function.

## Results

### Genome-wide mRNA profile of before and after LLDT-8 treatment

**Figure 2:**
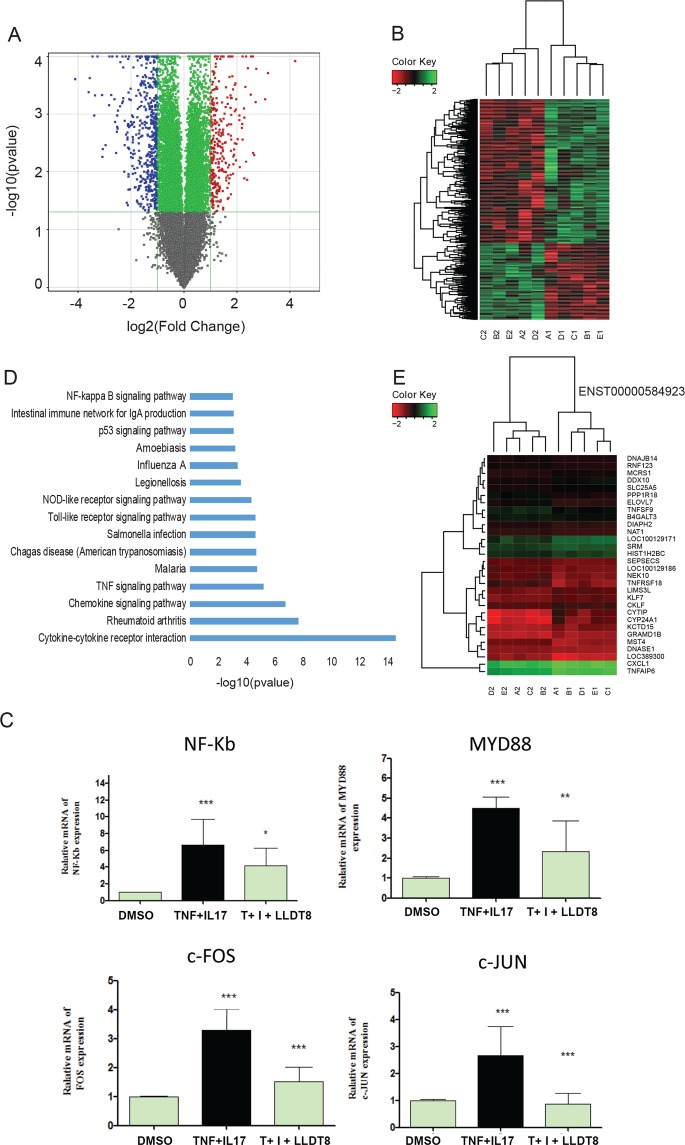
Genome-wide differential analysis for the treatment of LLDT-8 in FLS cells from RA patients. Volcano plot for the differential express genes. B. Heatmap plot for differential expressed genes to show the effect of LLDT-8 to FLS cells. C. qPCR validation to significant differential expressed genes from microarray. D. Gene ontology analysis to differential expressed genes (N = 3 technical repeats in control, TNF-α and LLDT8 treatment group). TNF-α treatment is applied to simulate inflammatory reaction and then LLDT8 treatment is applied to check the effect of LLDT8 to inflammatory reaction. E. Heatmap plot to the co-expressed mRNA of differential lncRNA have the power to separate samples with or without LLDT-8 treatment.

**Figure 3:**
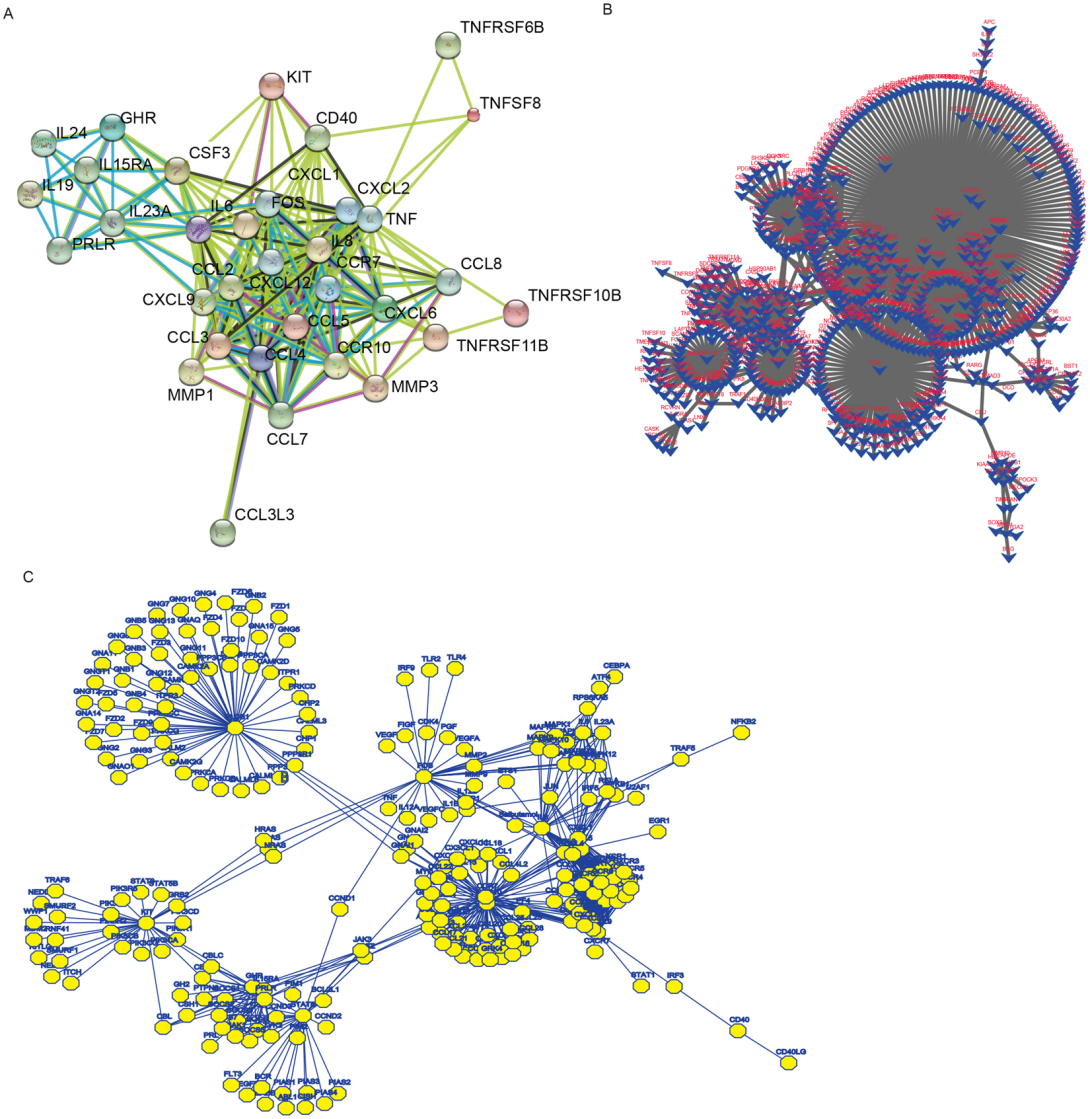
Interaction network analysis to differential expression genes based on STRING, BioGRID and KEGG. (**A**) Evidence view with STRING 9.0, (**B)** BioGRID based Network, (**C**) KEGG based network.

We describe the project with the flowchart as Fig. 1. By analyzing genome-wide lncRNA microarray of RA FLS responding to LLDT-8, we found 394 differentially expressed genes (p < 0.05, fold change > 2). 71% (281) of them were down-regulated and 28.7% (113) were up-regulated (Supp. Table 1). Volcano plot for the differential express genes was shown in Fig. 2A. Supervised cluster analysis (Heatmap) showed differentially expressed genes could distinguish the samples before and after LLDT-8 treatment (Fig. 2B). In order to check the quality of the microarray data, we validated several canonical immune responses relate genes, such as *NFKB1* (P = 1.80 × 10^−10^), *MYD88* (P = 1.26 × 10^−25^), *JUN* (P = 1.09 × 10^−14^) and *FOS* (P = 4.41 × 10^−22^) with qPCR and we found the result were highly consistent with microarray results (Fig. 2C). These down-regulated T-cell receptor signaling pathway (NFKB1, JUN, FOS) and Toll-like receptor signaling pathway (MYD88) genes indicate LLDT-8 could provide significantly immunosuppressive activity with multiple molecular networking approaches. KEGG pathway analysis (Fig. 2D) of the differentially expressed genes showed 20 pathways were significantly enriched (P < 0.05, FDR < 0.01). Interesting, the top 4 enriched pathways were significantly relevant to Immune reaction, including cytokine-cytokine receptor interaction (P = 4.61 × 10^−13^), rheumatoid arthritis (P = 1.90 × 10^−6^), chemokine signaling pathway (P = 1.01 × 10^−5^) and TNF signaling pathway (P = 2.79 × 10^−4^). These findings indicated that the LLDT-8 would greatly influence the RA FLS in the process of immune regulation. By analyzing interaction networks based on STRING (Fig. 3A), BioGRID (Fig. 3B) and KEGG (Fig. 3C), we found that these differentially expressed genes were significantly interacting with each other and constructed specific and explicit networks (Fig. 3).

### Genome-wide lncRNA profile of RA FLS before and after LLDT-8 treatment

The lncRNAs with both fold change ≥ 2.0 and P-value ≤ 0.05 from the *t*-test were identified as differentially expressed lncRNAs. Our results showed that 360 lncRNAs of RA FLS were significantly changed with LLDT-8 treatment. Those P-values ranged from 4.88 × 10^−2^ to 2.92 × 10^−7^, and the fold change spanned from 2.00 to 18.44. Among these lncRNAs, 56% (203) were downregulated and 44% (157) were upregulated (Supp. Table 2). Our result indicates not only mRNAs but also lncRNA transcripts would be widely changed during the treatment of LLDT-8. In addition, we found the target genes of the differential lncRNAs that could also be a strong indication of the change caused by LLDT-8 treatment. In addition, as Fig. 2E shows, cluster analysis based on the 30 co-expression genes of differential lncRNA (ENST00000584923) could separate the samples quite accurately with or without LLDT-8 treatment, indicating that the lncRNA network and the mRNA network in the cells were highly interacted and coordinately changed by LLDT-8 treatment.

### Co-expression network analysis between mRNA and lncRNA

The IncRNAs co-expressed function was identified through the correlation of lncRNA-mRNA. For each of the lncRNA, we calculate the Pearson correlation of its expression value with the expression value from every different mRNA. The pair of IncRNA and mRNA was selected if their correlation test reached the threshold (P-value < 0.05). Also, in order to ensure the selected pairs is meaningful, we only kept those correlated pairs which show strong negative correlation (correlation coefficient < −0.9). Our result indicated that there were 9,666 pairs of lncRNA-mRNA with strong negative correlation. Moreover, 618 of them were also identified as the significantly differential mRNA and lncRNA from the t-test in which 70 pairs of them were located in the same chromosome. Furthermore, 13 out of those 70 pairs were located in chromosome-6, and 10 out of those them were located in chromosome-17. Such kind of chromosome-distribution disequilibrium suggests the influence of the LLDT-8 is not a random effect but with specific target and influence to FLS. 27 cis-regulated (Table 2 and Supp. Table 3) and 591 trans-regulated lncRNA-mRNAs modules were identified (Table 3 and Supp. Table 4).

**Table 2:** Most significant trans-lncRNA-mRNA regulation pairs for the significant differential lncRNA.

lncRNA	Coordination	P	FC	Regulation	Gene Symbol	P-value	FC	Regulation	P	R
*FR264384*	chr9:2653919–2654254	1.03 × 10^−2^	2.67	down	*NINJ1*	2.30 × 10^−5^	2.4	up	2.40 × 10^−4^	−0.9
*LINC00473*	chr6:166352947-166401527	1.86 × 10^−2^	2.48	down	*HSPA1B*	7.52 × 10^−4^	3	up	2.16 × 10^−4^	−0.9
*linc-C20orf111-BP*	chr20:42839626-42855166	4.99 × 10^−4^	2.02	up	*SPTLC3*	6.30 × 10^−4^	2	down	1.08 × 10^−4^	−0.9
*linc-DHX37-4*	chr12:127215097-127221919	6.24 × 10^−4^	2.73	up	*TENC1*	4.70 × 10^−4^	2.1	down	1.30 × 10^−4^	−0.9
*linc-SCYL1-2*	chr11:65222664-65234212	2.97 × 10^−3^	2.56	up	*FTH1*	2.82 × 10^−4^	1.9	down	1.51 × 10^−4^	−0.9
*linc-SLC35F5-2*	chr2:114579399-114647368	3.80 × 10^−3^	2.17	up	*EHD3*	1.13 × 10^−3^	2.7	down	2.85 × 10^−4^	−0.9
*linc-TAGAP-1*	chr6:160007988-160015671	2.43 × 10^−6^	5.10	up	*GJA1*	1.72 × 10^−3^	2.5	down	2.78 × 10^−4^	−0.9
*PRLR*	chr5:35048860-35118224	1.50 × 10^−3^	3.46	up	*SPINK13*	1.75 × 10^−3^	2.6	down	2.38 × 10^−4^	−0.9
*RP11-356I2*.*4-006*	chr6:138175999-138179185	1.66 × 10^−4^	6.64	up	*PRDM1*	1.77 × 10^−3^	1.9	down	1.06 × 10^−4^	−0.9
*RP11-809O17*.*1-002*	chr8:142136390-142140060	2.84 × 10^−3^	2.09	up	*PNMA2*	1.46 × 10^−3^	2.4	down	2.51 × 10^−4^	−0.9
*SLC16A7*	chr12:60083117-60183635	9.31 × 10^−4^	2.34	down	*VAMP1*	2.01 × 10^−5^	2.3	up	1.53 × 10^−4^	−0.9
*SNORD3A-001*	chr17:19091329-19092027	4.55 × 10^−5^	11.0	down	*CCR10*	5.00 × 10^−5^	2.8	up	2.17 × 10^−4^	−0.9
*SNORD3A-001*	chr17:19091329-19092027	4.55 × 10^−5^	11.0	down	*HEXIM2*	3.21 × 10^−3^	2.1	up	2.88 × 10^−4^	−0.9
*SPECC1L-ADORA2A*	chr22:24666784-24838328	4.18 × 10^−5^	2.80	up	*TIMP3*	9.58 × 10^−4^	2.2	down	1.09 × 10^−4^	−0.9
*TNFRSF10B*	chr8:22877647-22926700	5.39 × 10^−4^	2.14	up	*PLEKHA2*	2.59 × 10^−3^	3.3	down	1.90 × 10^−4^	−0.9
*TNFRSF10B*	chr8:22877647-22926700	5.39 × 10^−4^	2.14	up	*SNTB1*	9.75 × 10^−6^	3.4	down	1.12 × 10^−4^	−0.9
*WISP1*	chr8:134203281-134243932	1.26 × 10^−4^	5.31	down	*SLCO5A1*	3.70 × 10^−4^	3.6	up	1.72 × 10^−4^	−0.9

Footnote: Coordination is based on GRCh37. P-value^A^ indicate the significance of the differential expression while P-value^B^ indicate the significance of the correlation between lncRNA and gene expression. Abbreviation: FC, Fold change; R, Correlation coefficient.

**Table 3:** Most significant cis-lncRNA-mRNA regulation pairs for the significant differential lncRNA

lncRNA	Coordination	P-value^A^	FC	R	Gene Symbol	P-value	FC	Regualation	P-value^B^	R
*FR407458*	chr7:100770384-100771815	1.82 × 10^−2^	2.1	down	*TIAM2*	6.42 × 10^−4^	2.15	up	1.01 × 10^−4^	−0.9
*AC005682*.*8-001*	chr7:22928990-22980809	2.71 × 10^−3^	4.1	up	*KIAA0226L*	2.17 × 10^−3^	3.25	down	1.01 × 10^−4^	−0.9
*PLCB1-IT1-001*	chr20:8229372-8237564	7.08 × 10^−3^	2.1	down	*HSPA1A*	3.44 × 10^−4^	3.02	up	1.01 × 10^−4^	−0.9
*CXCL1*	chr4:74735108-74737019	4.92 × 10^−5^	2.6	down	*TNFRSF18*	1.47 × 10^−3^	4.59	up	1.01 × 10^−4^	−0.9
*RP11-356I2*.*4-006*	chr6:138175999-138179185	1.66 × 10^−4^	6.6	up	*CYP24A1*	7.06 × 10^−5^	9.65	down	1.03 × 10^−4^	−0.9
*NKX3-1*	chr8:23536205-23540434	5.49 × 10^−4^	3.9	up	*G0S2*	3.05 × 10^−4^	2.84	down	1.03 × 10^−4^	−0.9
*FR018579*	chr10:63664416-63664655	1.13 × 10^−2^	2.3	down	*NEK10*	1.45 × 10^−3^	2.21	up	1.03 × 10^−4^	−0.9
*HNRNPDL*	chr4:83343716-83351378	7.53 × 10^−3^	2.1	down	*N4BP2L1*	6.45 × 10^−4^	2.17	up	1.03 × 10^−4^	−0.9
*FR066129*	chr4:74608854-74609068	1.11 × 10^−4^	4.3	down	*PHLDA2*	2.28 × 10^−4^	3.3	up	1.04 × 10^−4^	−0.9
*RP11-356I2*.*4-006*	chr6:138175999-138179185	1.66 × 10^−4^	6.6	up	*CCL8*	2.48 × 10^−4^	17.2	down	1.04 × 10^−4^	−0.9
*TRIB2*	chr2:12856997-12882858	5.35 × 10^−3^	2	down	*LOC727916*	2.36 × 10^−3^	3.41	up	1.04 × 10^−4^	−0.9
*FR264384*	chr9:2653919-2654254	1.03 × 10^−2^	2.7	down	*FAM129A*	6.45 × 10^−3^	2.08	up	1.04 × 10^−4^	−0.9
*linc-TRIM29-3*	chr11:121899038-121908990	9.03 × 10^−4^	2	up	*G0S2*	3.05 × 10^−4^	2.84	down	1.05 × 10^−4^	−0.9
*linc-DHX37-4*	chr12:127215097-127221919	6.67 × 10^−4^	3.2	up	*KIAA0226L*	2.17 × 10^−3^	3.25	down	1.05 × 10^−4^	−0.9
*RP11-356I2*.*4-006*	chr6:138175999-138179185	1.67 × 10^−4^	6.6	up	*LXN*	2.68 × 10^−4^	2.83	down	1.05 × 10^−4^	−0.9

Footnote: Coordination is based on GRCh37. P-value^A^ indicate the significance of the differential expression while P-value^B^ indicate the significance of the correlation between lncRNA and gene expression. Abbreviation: FC, Fold change; R, Correlation coefficient.

Functional prediction of selected differential lncRNAs analysis were conducted both with GO and KEGG strategies. Gene Ontology analysis showed that the term of innate immune responses was significantly enriched and 17 differential lncRNAs were identified to be involved (Supp. Table 5). KEGG analysis identified term of rheumatoid arthritis was significant enriched and 4 differential lncRNAs (NONHSAG028996, NONHSAT142637, ENST00000584934 and NR_028330.1) were identified to be involved (Supp. Table 6).

**Figure 4:**
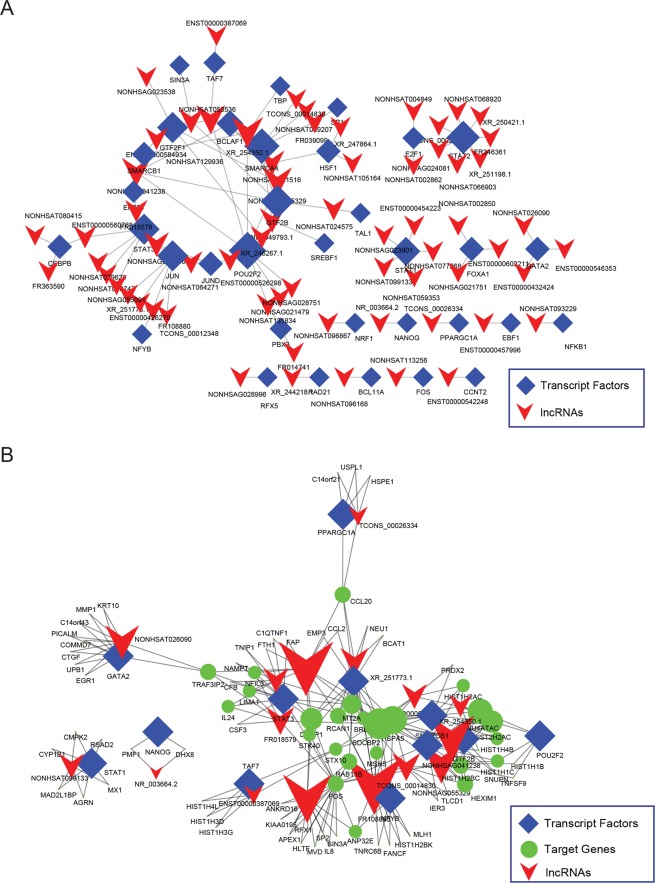
Co-expression network of lncRNA-TF and lncRNA-TF-targetGene within LLDT-8 differential response transcriptional regulatory network. (**A**) Co-expression network of lncRNA-TF, (**B**) Co-expression network of lncRNA-TF-Target Genes.

We also identified large number of co-expressed lncRNA and TFs. The co-expression network between TF and lncRNA was shown in Fig. 4A. We discovered one large sub-network and large number isolated interaction between TFs and lncRNA. This network indicated lncRNA and TF would regulate the gene expression together in FLS cells. When we build lncRNA, TF, target gene network together (Fig. 4B), the result is quite similar as our expectation, and large numbers of genes were identified regulated by lncRNA and TF simultaneously.

### Cellular regulation effect of LLDT-8 treatment to FLS involved in NF-κB pathway

**Figure 5:**
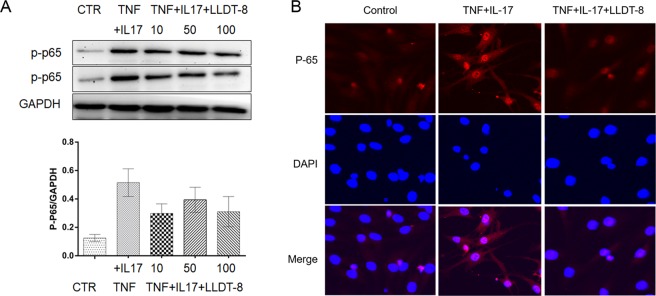
Western Blot and Immunofluorescence to show LLDT-8 effect on p-P65 protein gene expression and nuclear transform. (**A**) Western blot to show the suppressive functions of LLDT-8 to p-p65 protein in different dose (10 ng/ml, 50 ng/ml and 100 ng/ml). (**B**) Immunefluorescence and confocal microscopy to show the suppressive function of LLDT-8 to the nuclear transform of p-p65.

In order to validate our discoveries that LLDT-8 could provide pharmacological effect to RA therapy via the interaction with rheumatology related pathway (Fig. 2D), we validate the effect of LLDT-8 to one of most important rheumatology related pathway, NF-kappa B signaling pathway. Western-blot shown that the protein level of p-p65 are strongly inhibited with increasing concentration of LLDT-8 treatment to FLS cells stimulated by TNF-α and IL-17 (Fig. 5A). In addition, we also demonstrate that LLDT-8 would inhibit the nuclear translocation of the p65 (Fig. 5B) which is the significant effect of TNF and IL-17, indicating LLDT-8 has significant inhibition to TNF and IL-17 effects and therefore have potential pharmacological effect to RA therapy.

## Discussion

*Tripterygium* is a traditional Chinese medicine, which has widely been used in the treatment of rheumatic disease. (5R)-5-hydroxytriptolide (LLDT-8) is an extracted compound from *Tripterygium* and has been showed lower cytotoxicity and relatively higher immunosuppressive activity. However, how LLDT-8 influences RA FLS cells is still unknown, especially in the level of lncRNAs. In this study of genome-wide microarray assay, we identified large number of lncRNA and mRNAs responding to the LLDT-8 treatment. In addition, the first LLDT-8 related cis-regulated and trans-regulated lncRNA-mRNA modules were identified. We also constructed the first lncRNA-TF-mRNA co-expression network and demonstrated that LLDT-8 influenced the expression network within the whole FLS cells and therefore provided potential molecular and cellular mechanisms that LLDT-8 could be considered as potential rheumatoid arthritis drugs.

As we know, this is the first study to elaborate the genome-wide lncRNA and mRNA changes with or without LLDT-8 treatment. Our study provided an important landscape to design further studies to investigate the medical applications for lncRNAs in RA therapy. Although, genome-wide association studies (GWAS) have identified hundreds of variants associated with RA, our understanding of the disease mechanisms is still limited. Notably, more than 90% of the risk variants lie in non-coding regions, and almost 10% are corresponding to lncRNA regions^23,35,36^. Considering that our result showed the significant change of the lncRNA during LLDT-8 treatment, lncRNA would be play important role in the pathogenesis and therapy of RA.

Our microarray data are consistent with protein results in our previous study in which we found LLDT-8 inhibited IL-1β, IL-6, IL-21 and IL-23 while LLDT-8 increased the IL-10 protein level. Meanwhile, our research were supported by numerous of previous research, such as Dr. Karouzakis and colleagues found synovial fibroblasts from early rheumatoid arthritis shown DNA methylation changes^37^. Our research demonstrate the immunosuppressive function of LLDT-8 was related to the interaction with ncRNA network. Our results were also consistent with n’sh (PMC2833606) in which they found CCL8 are abundantly-expressed in RA synovial tissue while we found LLDT-8 could significant decrease CCL8 (Table 3). In another study^38^, p38 MAPK inhibition treatment showed increased TNFRSF18 which is similar with LLDT-8 treatment in our study which also highlights the anti-inflammatory function of LLDT-8^38^. What’s more, FAM129A was demonstrated low-expression in RSK2 deficiency mice which have earlier and exacerbated inflammation of bone destruction and in our study, we found FAM129A was significantly up-regulated after LLDT-8 treatment^39^. All these evidence support our conclusion that LLDT-8 own immune-suppressive function and epigenetic regulation mediated by lncRNA might play important roles.

We conducted the power analysis to estimate the minimum samples size to identify significant (q-value < 0.5) mRNA changes based on our dataset GSE84074 with bootstrap resampling. In the bootstrap resampling, we selected several important rheumatoid arthritis genes such as MMP genes (*MMP1*, *MMP3*), CCL genes (*CCL3*, *CCL4*, and *CCL26*), and IL genes (*IL6*, *IL8*, *IL19*, and *IL24*). We found when the sample size is larger than 4, majority of these rheumatoid arthritis genes can be detected with power > 0.8 (Supplementary Fig. 2), therefore, we believe our sample size is sufficient to identify the most interesting inflammation and autoimmune associated signals.

Our study also provided a probability to compare the lncRNA profiles with RA, primary Sjögren’s syndrome^40^, gastric carcinogenesis^41^, endometrial carcinoma^42^, bladder cancer^43^, esophageal squamous cell carcinoma^44^, non-small-cell lung cancer^45^ and cervical cancer^46^, since our data has been deposited to GEO database. These diseases might share some common aberrant lncRNAs relevant with inflammation and immune response and therefore provide some special insight to the therapy of RA.

In summary, our results provided a good prospect in further clinical tests of LLDT-8 for its therapeutic potential in the treatment of RA. We also suggest that more studies need to be done in order to further investigate the biological functions of IncRNAs in more patient samples. However, those initial results suggest that lncRNA would be a good biomarker and drug target in the future drug discovery, especially for LLDT-8. Our study also demonstrate epigenetics including DNA methylation^47–49^ and lncRNA^50,51^ are playing important roles in etiology and therapy of RA.

## Conclusion

Genome-wide lncRNA and transcriptome analysis of response to treatment with (5R)-5-Hydroxytriptolide (LLDT-8) in cells shown LLDT-8 would mainly influence the RA cells systemically and especially in the process of immune network regulation.

## Supplementary information

SUPPLEMENTARY INFO

Supp. Table 1

Supp. Table 1

Supp. Table 3

Supp. Table 4

Supp. Table 5

Supp. Table 6

## Data Availability

The microarray data was deposited in the Gene Expression Omnibus (GEO accession: GSE84074).
