# Supplementary material for: (5R)-5-Hydroxytriptolide (LLDT-8) induces substantial epigenetic mediated immune response network changes in fibroblast-like synoviocytes from rheumatoid arthritis patients
Source: Sci Rep. 2019 Aug 1;9:11155. doi: 10.1038/s41598-019-47411-1 (PMC6671973; doi:10.1038/s41598-019-47411-1)
Supplement: Supplementary file 1 — SUPPLEMENTARY INFO [file 41598_2019_47411_MOESM1_ESM.docx]

**(5R)-5-Hydroxytriptolide (LLDT-8) induces substantial epigenetic mediated immune response network changes in fibroblast-like synoviocytes from rheumatoid arthritis patients**

Shicheng Guo^1#^, Jia Liu^2,3#^, Ting Jiang^2,3^, Dungyang Lee^4^, Rongsheng Wang^2,3^, Xinpeng Zhou^2^, Yehua Jin^2^, Yi Shen^2,3^, Yan Wang^3^, Fengmin Bai^2,3^, Qin Ding^2,3^, Grace Wang^5^, Jianyong Zhang^6^, Xiaodong Zhou^7^, Steven J. Schrodi^1,8^, Dongyi He^2,3$^

^1^Center for Precision Medicine Research, Marshfield Clinic Research Institute, Marshfield, WI, United States, 54449

^2^Department of Rheumatology, Shanghai Guanghua Hospital of Integrated Traditional and Western Medicine, Shanghai 200052, China.

^3^Arthritis Institute of integrated Traditional and Western medicine, Shanghai Chinese Medicine Research Institute, Shanghai 200052, China.

^4^Division of Biostatistics, University of Texas School of Public Health, Houston, TX, USA

^5^Washington University, St. Louis, Missouri, USA. 63130

^6^Shenzhen Traditional Chinese Medicine Hospital and The fourth Clinical Medical College of Guangzhou University of Chinese Medicine. Fuhua Road, Shenzhen, Guangzhou, China, 518033

^7^University of Texas Medical School at Houston, 6431 Fannin, MSB5.270, Houston, TX 77030, USA

^8^Computation and Informatics in Biology and Medicine, University of Wisconsin-Madison, Madison, WI 53706, USA

# Correspondence:

Dr. Dongyi He

[dongyihe@medmail.com.cn](mailto:dongyihe@medmail.com.cn)


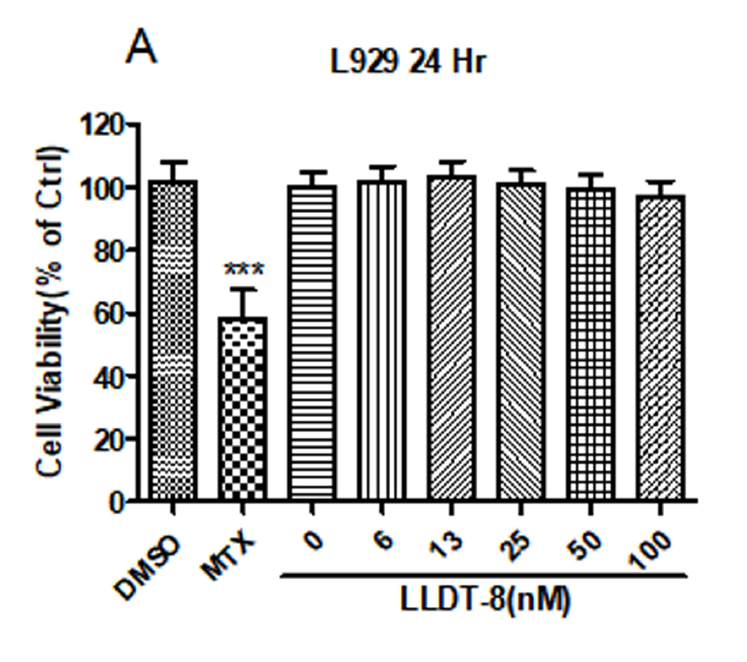


**Supplementary Figure 1**. CCK-8 and apoptosis assay to show the cytotoxic and apoptosis effect of LLDT-8 to RA-FLS cells. A. CCK-8 assay to show LLDT-8 cytotoxic effect in different dose from 0nm/ml to 100nm/ml.


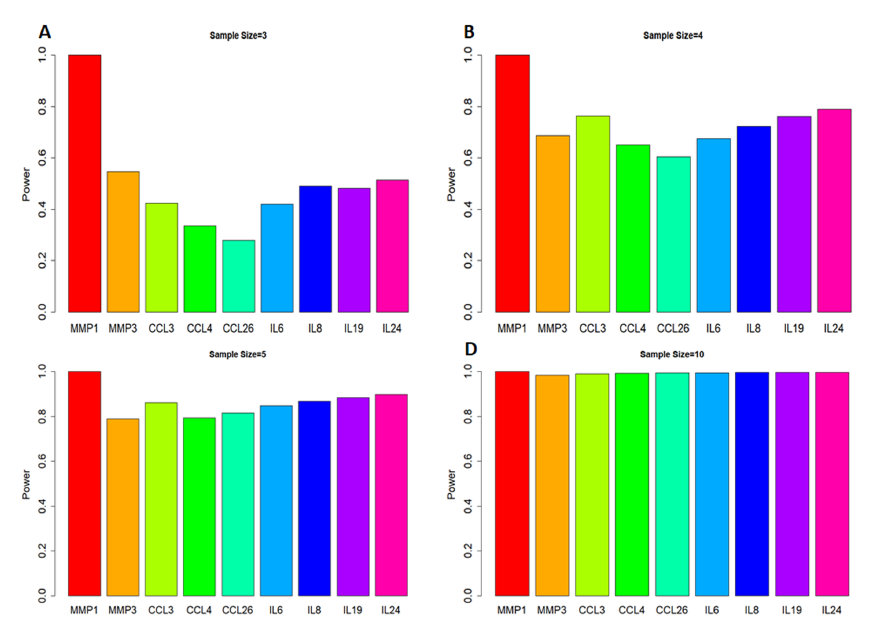


**Supplementary Figure 2.** Power analysis to estimate the minimum samples size to identify significant (q-value <0.5) mRNA changes based on our dataset [GSE84074](https://www.ncbi.nlm.nih.gov/geo/query/acc.cgi?acc=GSE84074) with bootstrap resampling
